# From theory to practice: understanding the challenges in the implementation of electrogenerated chemiluminescence for analytical applications

**DOI:** 10.1007/s00604-024-06413-1

**Published:** 2024-05-31

**Authors:** Gabriele Giagu, Alessandro Fracassa, Andrea Fiorani, Elena Villani, Francesco Paolucci, Giovanni Valenti, Alessandra Zanut

**Affiliations:** 1 Department of Chemistry Giacomo Ciamician, University of Bologna, via Selmi 2, Bologna, 40126 Italy; 2 Department of Chemistry, Keio University, 3-14-1 Hiyoshi, Yokohama, 223-8522 Japan; 3 Department of Chemical Science and Engineering, School of Materials and Chemical Technology, Tokyo Institute of Technology, Yokohama, 226-8502 Japan; 4 Present Address: Department of Chemical Sciences, University of Padova, via Marzolo 1, Padua, 35131 Italy

**Keywords:** Electrochemiluminescence, Biosensor, Electrochemistry, Sensors

## Abstract

Electrogenerated chemiluminescence (ECL) stands out as a remarkable phenomenon of light emission at electrodes initiated by electrogenerated species in solution. Characterized by its exceptional sensitivity and minimal background optical signals, ECL finds applications across diverse domains, including biosensing, imaging, and various analytical applications. This review aims to serve as a comprehensive guide to the utilization of ECL in analytical applications. Beginning with a brief exposition on the theory at the basis of ECL generation, we elucidate the diverse systems employed to initiate ECL. Furthermore, we delineate the principal systems utilized for ECL generation in analytical contexts, elucidating both advantages and challenges inherent to their use. Additionally, we provide an overview of different electrode materials and novel ECL-based protocols tailored for analytical purposes, with a specific emphasis on biosensing applications.

**Graphical abstract:**

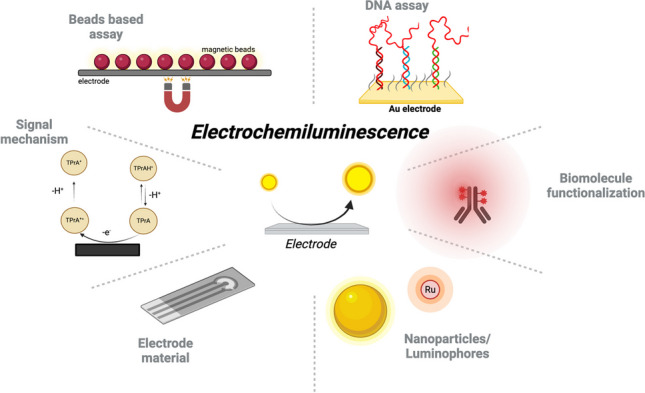

## Introduction

Electrogenerated chemiluminescence (ECL) is a phenomenon of light emission at electrodes in electrochemical cells caused by energetic electron transfer (redox) reactions of electrogenerated species in solution [1, 2]. The peculiarity of this technique lies in the generation of reactive intermediate species at the electrode’s surface that react with one another, resulting in an excited state capable of emitting light [3]. This way of generating excited states in ECL makes it an extraordinarily versatile technique ensuring remarkable sensitivity and nearly absent background optical signals. This is attributed to the activation of luminophores through electrochemical processes rather than external light stimuli.

In addition to that, by tuning the applied voltage or current, the rate of electron transfer reactions can be precisely controlled, thereby affecting the kinetics of the overall reaction. This capacity for fine-tuning enables the optimization of reaction conditions, leading to improved reproducibility, efficiency, and selectivity in the ECL process.

ECL benefits also from a diverse array of molecules that can act as luminophores, along with compatible coreactants. This extensive selection ensures optimal compatibility across various systems, including aqueous solutions. Moreover, the integration of electrochemiluminescent labels with biological molecules enables the development of highly sensitive and specific biosensors allowing researchers to design assays tailored to specific applications, thereby enhancing the precision and efficiency in detecting and analyzing biomolecular interactions [4–6].

For instance, in biosensing, ECL is used for detecting and quantifying a wide range of biomolecules with outstanding sensitivity and specificity, making it useful for applications in medical diagnostics, environmental monitoring, and drug development. On the other hand, in imaging applications, ECL emerges as a formidable tool for visualizing biological processes at both cellular and molecular scales. This capability provides researchers with a powerful tool to investigate physiological mechanisms and understand disease pathology [7–10].

In ECL, the excited state capable of emitting light is generated through a combination of electrochemical and chemical reactions which can be classified in two main categories: annihilation mechanism [11] and coreactant mechanism [12, 13].

The annihilation mechanism involves an electron transfer reaction between high energetic radical anion and cation of the luminophore, which are generated electrochemically at the electrode, with consequent population of the excited state. This is generally achieved by fast switching the electrode potential from oxidation to reduction currents (or vice versa), with radical annihilation taking place inside the diffusion layer [14].

The coreactant mechanism involves a chemical reaction that occurs after the electrogeneration of the radicals and before the electron transfer to excite the luminophore. The coreactant is a sacrificial molecule that conversely to the luminophore is not recovered after the light emission. It is, in fact, this irreversible chemical reaction of the coreactant (after its oxidation or reduction) that promotes the transformation of the coreactant into a high energetic radical specie [12, 15]. In the following sections, we aim to give useful information on the application of ECL in analytical settings. In particular, we will provide an overview of the theoretical foundations of ECL generation, and we will outline the key systems employed for ECL generation in analytical scenarios, discussing both their advantages and challenges.

## Energy requirements for light emission in ECL

The main focus of an ECL reaction centers around the generation of an excited species capable of emitting light, as previously noted.

In the annihilation reaction, the energy required for the formation of the excited state originates from the interaction between the two radical ions, which are produced by the alternating switch of the potential of the electrode between positive and negative values [16].

ECL emission can only occur if the combined energy of these two species exceeds that of the excited state [17] (Eq. 1).

1 $$\varDelta G={E}_{red}^{^\circ }-{E}_{ox}^{^\circ }+{E}_{es}$$

In this context, Δ*G* is the Gibbs free energy change for the annihilation reaction, *E*^*0*^_red_ and *E*^*0*^_ox_ are the standard potentials of the luminophore for reduction and oxidation, respectively, and *E*_es_ is the energy difference between the emitting excited state and the ground state of the luminophore [4].

Conversely, in a reaction involving a coreactant, the excited state arises from species generated via an electron transfer reaction between the luminophore (or one of its derivatives) and a species produced following the oxidation or reduction of the coreactant at the electrode surface [18]. For the coreactant pathway, the Δ*G* is as reported in Eq. 2 or 3 depending on the oxidized or reduced form of the luminophore, respectively:

2 $$\varDelta G={E}_{Cor}^{^\circ }-{E}_{Ox}^{^\circ }+{E}_{es}$$

or

3 $$\varDelta G={E}_{Red}^{^\circ }-{E}_{Cor}^{^\circ }+{E}_{es}$$

These equations exemplify a reaction where the ECL emission occurs through coreactant and luminophore electron transfer reaction. In this scenario, a highly reactive radical species originated from the coreactant generates the excited state, rather than the electro-reduced (or oxidized) form of the luminophore. Importantly, as Eqs. 2 and 3 suggest, successful light emission relies on the effective energy transfer facilitated by electron transfer (Δ*G* < 0). This implies a tight link between the electron transfer energy and the luminophore’s emission energy. Essentially, when the emission energy is high, it becomes essential to have a coreactant capable of providing the necessary energy [19, 20].

Such boundary conditions have been defined as ECL “wall of energy sufficiency,” and it could be implied to predict whether a luminophore is theoretically capable of emitting light.

**Fig. 1:**
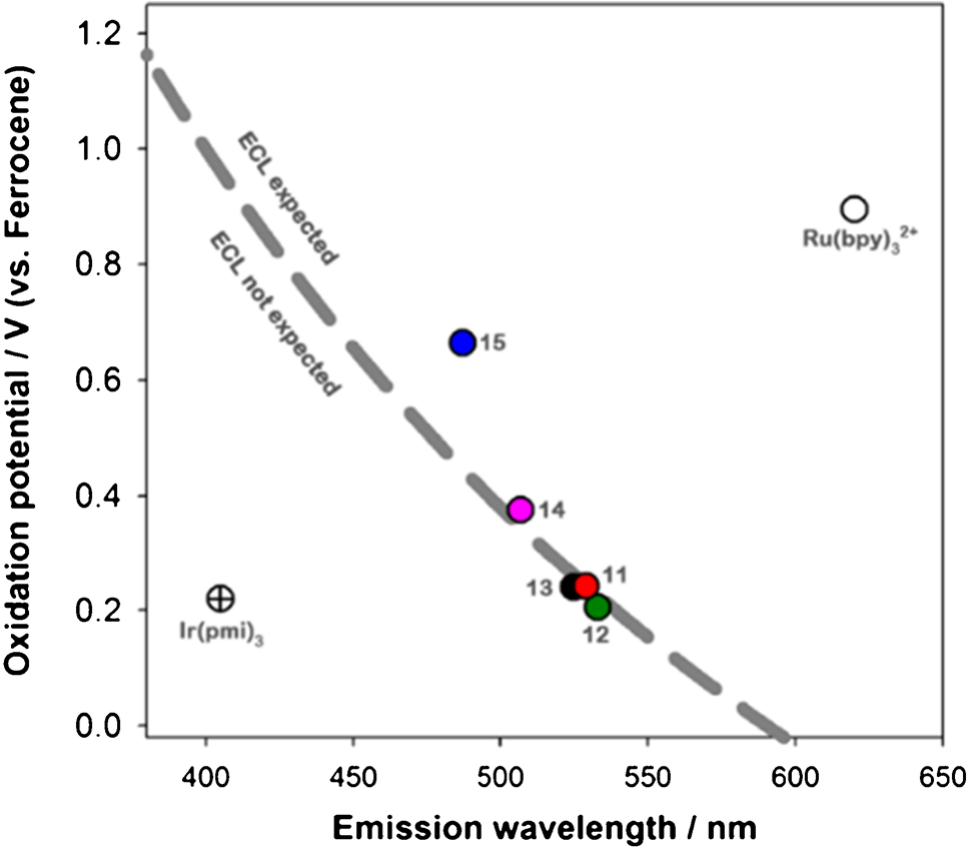
This plot allows to visualize the “wall of energy sufficiency” for a TPrA system; each point represents a luminophore; luminophores above the dotted line are expected to emit light after electron transfer reaction with TPrA^•^; the ones below are expected to not emit light. Numbers 11–15 indicate the different Ir(ppy)_2_ (C^C:) complexes tested. Adapted with permission from [21], Copyright (2014) American Chemical Society

Stringer et al. provide a compelling example of this study [21]. They compared various metal complexes as potential luminophores in the coreactant ECL system with TPrA through the catalytic route. Their analysis relied on a “wall of energy sufficiency” plot (Fig. 1) which correlates the oxidation potential of each metal complex with its emission wavelength, where each point represents a potential luminophore. The dotted line indicates the “wall of energy sufficiency” for the TPrA system.

**Fig. 2:**
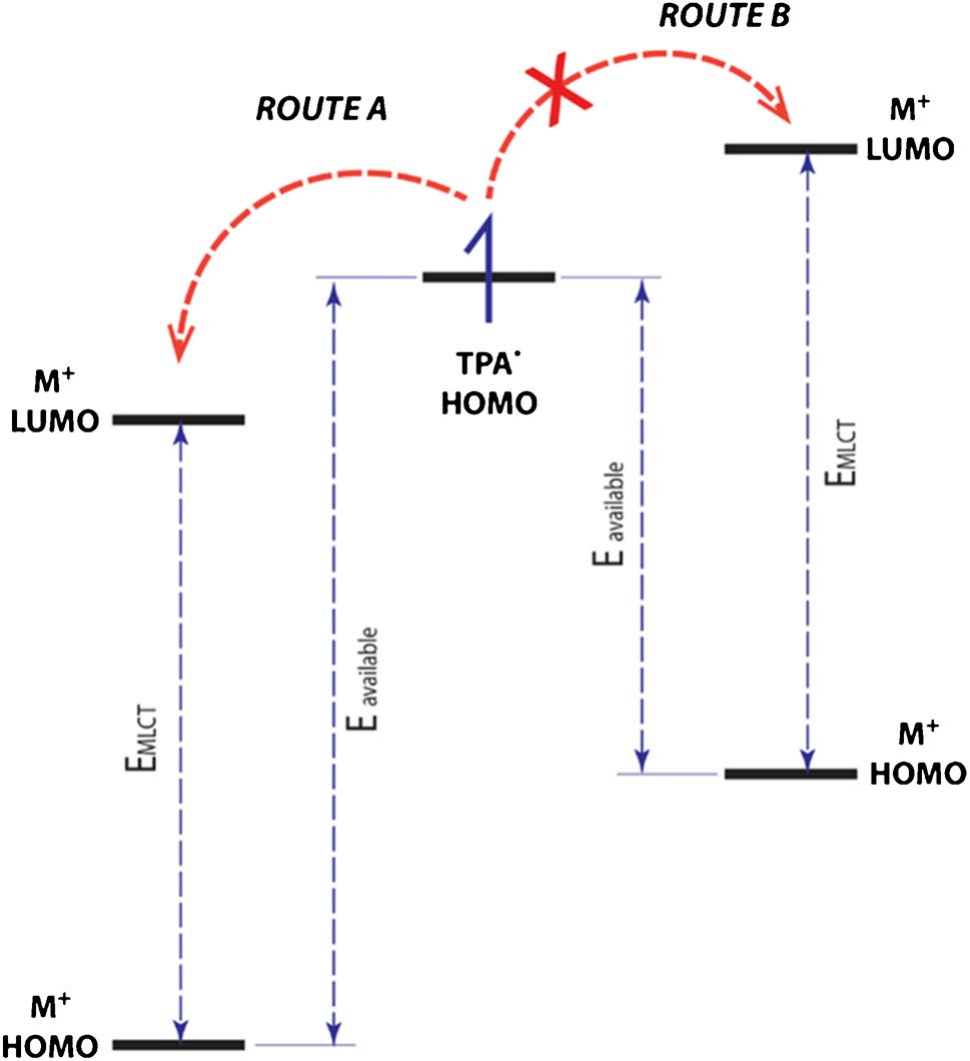
Schematic representation of the ET reaction between TPrA^•^ and the luminophore for energy sufficient ET reaction for the population of the excited state (pathway A) and for non-sufficient ET reactions (pathway B). Adapted with permission from [21], Copyright (2014) American Chemical Society

This scheme emphasizes the role of energy requirements to obtain a specific emission wavelength. A favorable luminophore must have an oxidation potential positive enough to enable efficient electron transfer between the HOMO state of TPrA and the LUMO state of the luminophore (Fig. 2).

The Marcus-Hush theory helps to explain why a more positive Δ*G* value favors the reaction leading to the excited state (inverted region) over the reaction leading to ground state products (normal region), based on kinetic aspects [22, 23].

When considering TPrA for ECL signal generation in a heterogeneous system (where the luminophore is fixed, as we will explore further), it is important to understand its mechanism introduced by Bard and his colleagues [12].

This mechanism differs in that the luminophore itself does not undergo an electrochemical reaction. Instead, TPrA acts as the sole species undergoing oxidation, forming the $\mathrm{TPrA}^{\bullet+}$ radical cation. This radical quickly decomposes into the $\mathrm{TPrA}^{\bullet}$ radical, which then reduces the luminophore. The resulting reduced luminophore can subsequently react with the $\mathrm{TPrA}^{\bullet+}$ radical cation, leading to the generation of the excited state.

The energy involved in this heterogeneous reaction can be expressed as:

4 $$\varDelta G={E}_{Red}^{^\circ }-{E}^{^\circ }\left({TPrA}^{\bullet+}\right)+{E}_{es}$$

**Fig. 3:**
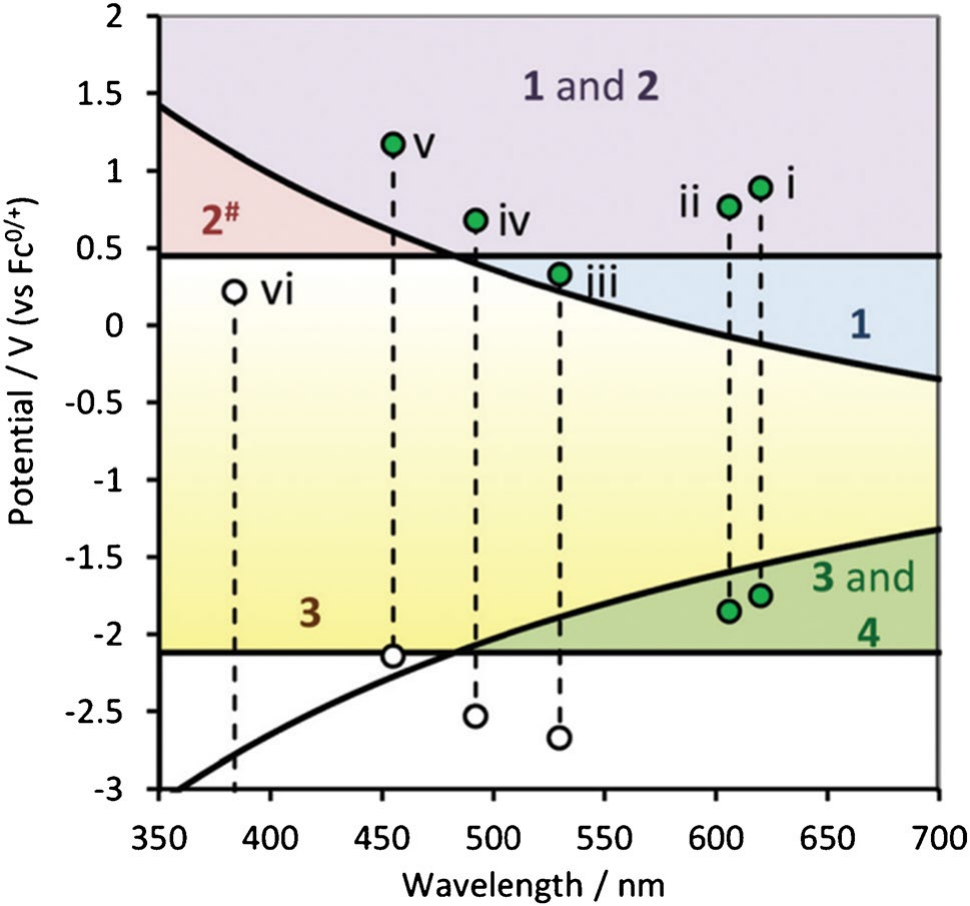
Complete “wall of energy sufficiency” plot representation for complexes in a TPrA coreactant system showing the energy requirements for the different reaction pathways between the luminophore and the coreactant. This diagram depicts the energy requirements for various metal complexes, considering both their redox potentials and emission wavelengths. The numbers within the zones indicate which of the four TPrA reaction pathways are available for each complex based on its energetic positioning. Adapted with permission from [24], Copyright (2016) American Chemical Society

This statement necessitates a critical consideration which involve an additional “energy barrier” that behaves similarly to the wall of energy (Fig. 3). In this case, for efficient electron transfer to populate the luminophore’s LUMO (lowest unoccupied molecular orbital), the reduction potential (${E}_{Red}^{^\circ }$) must be sufficiently negative. This ensures that the energy transferred from $\mathrm{TPrA}^{\bullet+}$ to the luminophore is sufficient to overcome the energy gap between its ground and excited states. Essentially, a “favorable” energy landscape is required for successful light emission in this heterogeneous pathway [24–26].

## ECL systems

ECL systems can be classified in several ways, for example based on (i) the luminophore [27], (ii) the coreactant [28], and (iii) the reaction mechanism that occurs between the two. Here, annihilation ECL will not be described as it is not relevant for analytical applications [6], although it holds important theoretical and mechanistic aspects [29].

Concerning the luminophores, there are inorganic complexes [3], mainly of Ru(II) [30] or Ir(III) [31, 32], luminol [33, 34], organic molecules (polycyclic aromatic hydrocarbons, BODIPY, fluorene, and spirobifluorene) [35, 36], carbon nanomaterials (carbon dots and graphitic carbon nitride) [37], semiconducting nanocrystals (NCs) and quantum dots (QDs) [38–42], and gold (Au) nanocluster [43].

However, among all the luminophores available, commercial applications of ECL exploit only ruthenium complexes, although iridium is gaining more attention for its promising higher photoluminescence and ECL efficiency than ruthenium [44]. Inorganic complexes offer a wide range of possible functionalization, are water soluble although iridium complexes require careful design to be made soluble while retaining efficient coreactant ECL [31], and can be synthetized homogeneously and precisely at the molecular level. Organic molecules have generally low or no water solubility; therefore, the conjugation synthesis to the biological receptor is complicated, or it requires expensive (cost and workup) modification of the luminophore. Nanomaterials are difficult to be produced in a standardized form to obtain the accuracy and reproducible results essential for applications actually reached in ECL analyzers.

In the group of coreactants, it is possible to find amines, the most efficient and used of those is tri-*n*-propylamine (TPrA), and oxalate for “oxidative-reduction” ECL mechanism (oxidation reaction), while persulfate, hydrogen peroxide, and benzoyl peroxide are suitable for “reductive-oxidation” ECL mechanism (reduction reaction). Other coreactants exist, namely amine-related coreactants (amino acids, peptides, nucleic acid, NADH, alkaloids, pharmaceuticals, pesticides, hydrazine), organic acids, and alcohols, although the ECL signal can be considerably low. However, these molecules can be detected directly by acting both as coreactant and analyte [45].

Generally, the luminophore reacts with the coreactant, and it is regenerated after the light emission, except for luminol that is converted to an unreactive compound, 3-aminophthalate dianion [33]. Therefore, a useful classification of the ECL systems can be done based on the reaction mechanism that is peculiar of each coreactant. In the following section, the most important ECL mechanisms are described, providing their possible application in sensor development.

### ECL mechanism by “oxidative-reduction”

This category comprises the coreactants that after oxidation form a high energetic radical that reduces the luminophore, such as oxalate and amines.

**Scheme 1:**
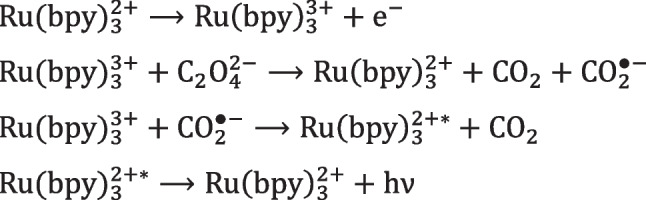
ECL mechanism of oxalate and Ru(bpy)_3_^2+^

Oxalate was the first coreactant developed for aqueous solution reacting with Ru(bpy)_3_^2+^ [46], and for its detection in synthetic urine [47], as reported in Scheme 1.

**Scheme 2:**
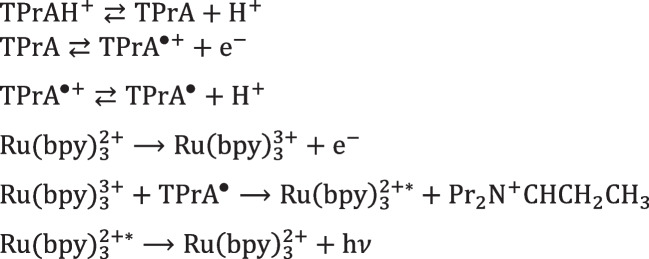
ECL “oxidative-reduction” mechanism of TPrA and Ru(bpy)_3_^2+^

**Scheme 3:**
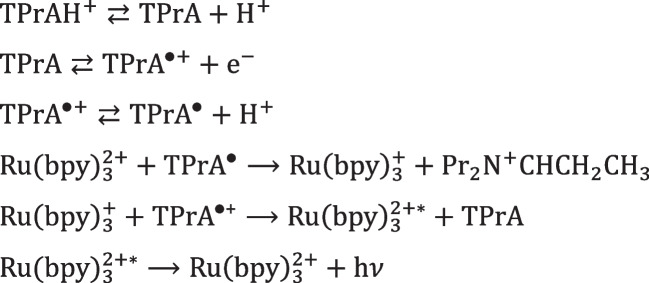
Heterogeneous ECL mechanism of TPrA and Ru(bpy)_3_^2+^

**Scheme 4:**
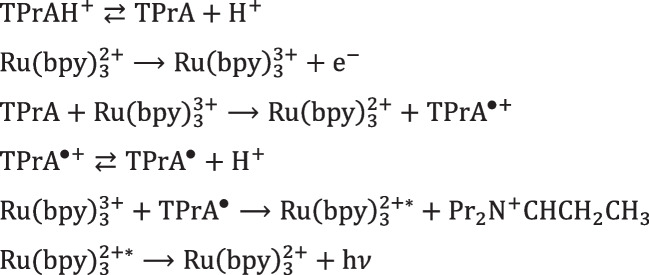
Catalytic ECL mechanism of TPrA and Ru(bpy)_3_^2+^

The mechanism with TPrA is more complex and offers a wider applicability making it the only available to develop commercial instruments for clinical analysis. Beside the first described “oxidative-reduction” mechanism (Scheme 2), a mechanism that involves exclusively TPrA oxidation (Scheme 3) and a catalytic mechanism (Scheme 4) occur. In addition, annihilation between Ru(bpy)_3_^3+^ and Ru(bpy)_3_^+^ is theoretically possible in presence of TPrA [12].

**Fig. 4:**
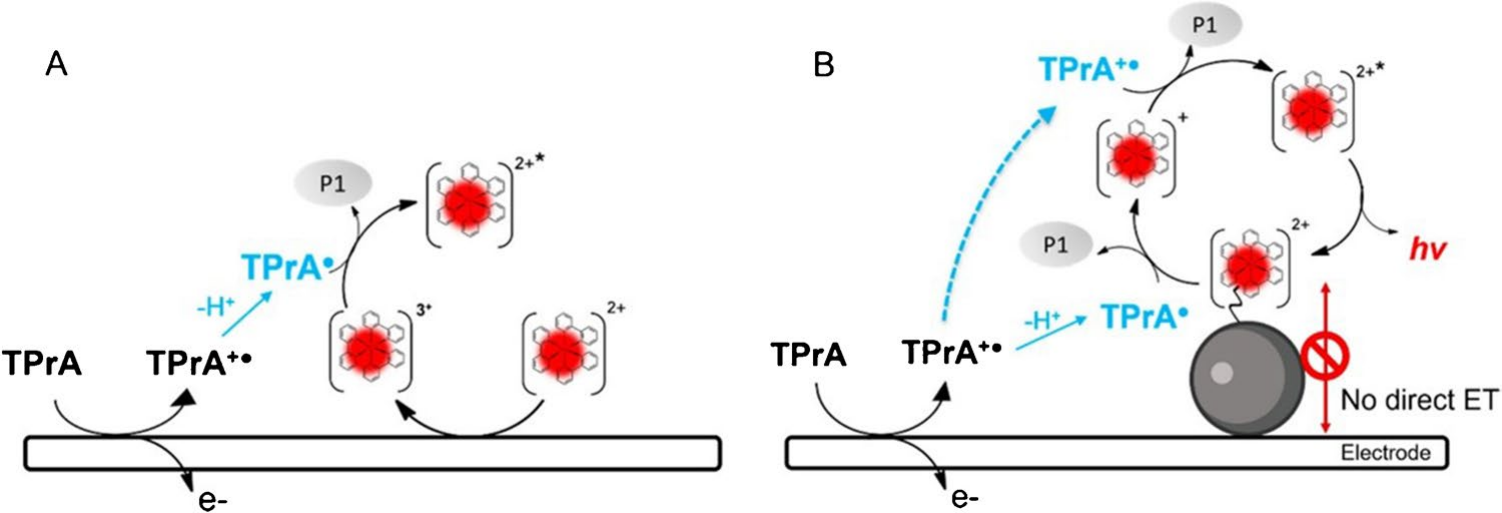
Schematic representation of electrogenerated chemiluminescence mechanism involving TPrA and Ru(bpy)_3_^2+^: (**a**) homogeneous, where both coreactant and luminophore can be oxidized at the electrode surface; (**b**) heterogeneous where only the coreactant is oxidized. Adapted with permission from [50], Copyright (2022) American Chemical Society

Scheme 2 reports the first mechanism ever investigated with TPrA in aqueous solution (Fig. 4A) [48] which opened the application to clinical analysis and its commercial development [49]. Only few years later, the real mechanism responsible of the ECL emission in clinical analyzer was presented in a seminal paper from the Bard group [12] that has been demonstrated to involve only TPrA oxidation (Fig. 4B), while direct oxidation of Ru(bpy)_3_^2+^ was not necessary (Scheme 3).

The nomenclature “heterogeneous” comes directly from the application in the ECL analyzers where the Ru(bpy)_3_^2+^ is immobilized on microbeads or directly onto the electrode [3, 6, 15], meaning that it is not free to diffuse because belonging on a different phase (Scheme 3, Fig. 4B), in contrast with homogeneous case where both Ru(bpy)_3_^2+^ and TPrA are freely to diffuse in solution (Scheme 2, Fig. 4A).

**Fig. 5:**
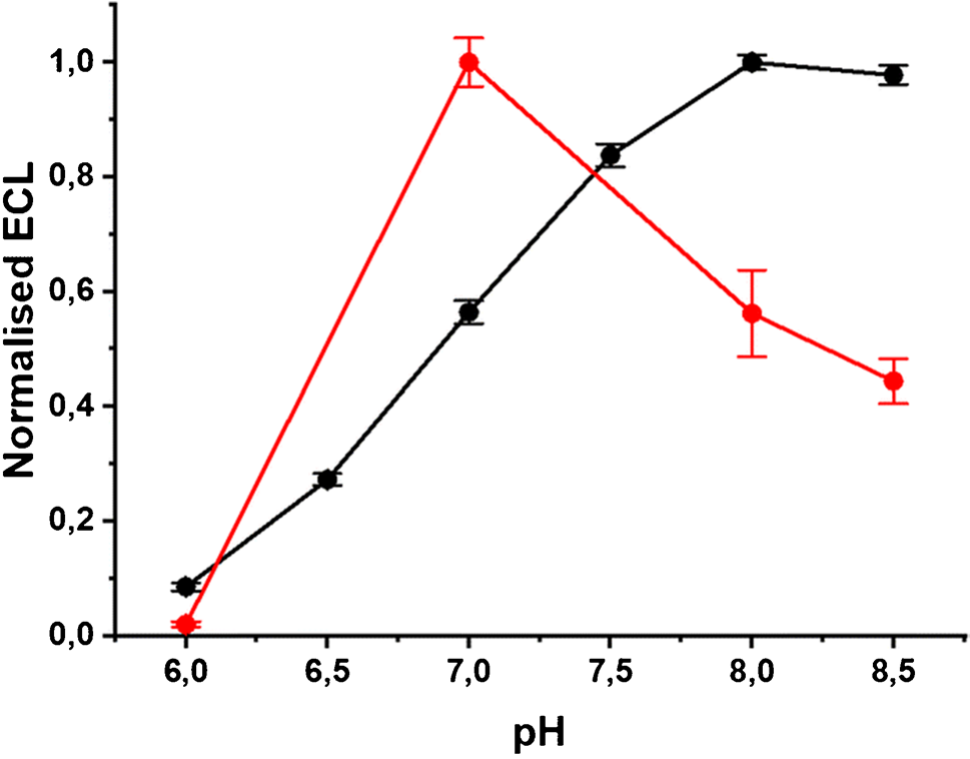
Normalized ECL as a function of pH: Ru(bpy)_3_^2+^ free diffusing in solution (black), and Ru(bpy)_3_^2+^ labeled on 2.8-µm beads deposited on the electrode (red). Adapted with permission from [51], Copyright (2023) Royal Society of Chemistry

The difference in the ECL mechanisms is highlighted in the different optimal pH value for the highest ECL emission (Fig. 5).

In fact, the reaction rate of the overall ECL mechanism is affected by the TPrA radical cation deprotonation to form the TPrA radical which is a function of the pH. Faster deprotonation in high pH increases the rate of mechanism 2 (Fig. 4A), while in low pH, the slower deprotonation enables the heterogeneous ECL mechanism 3 to be quantitatively relevant for the signal emission [52].

Beside the pH effect, the effect of amine radical cation deprotonation was clearly evident with the use of 2-(dibutylamino)ethanol (DBAE), in comparison with TPrA. The faster deprotonation enables high ECL signal for the mechanism 2 [53], but the emission is almost suppressed for heterogeneous ECL mechanism 3 when used with microbeads (Fig. 4B) [8].

The last mechanism that is possible to occur with the Ru(bpy)_3_^2+^/TPrA system is the catalytic mechanism, where the freely diffusing Ru(bpy)_3_^3+^ can oxidize the TPrA in solution as represented in Scheme  4.

**Fig. 6:**
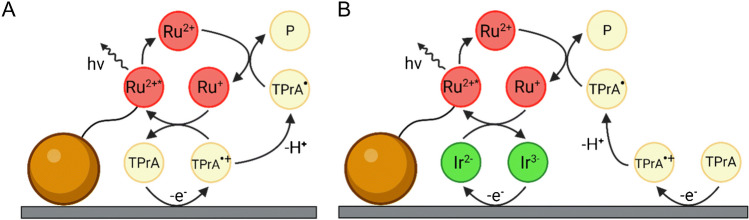
Schematics of heterogeneous bead-based immunoassay: conventional ECL pathway (**a**) and the enhanced “redox mediated” pathway (**b**). The magnetic microbead is represented by the orange sphere while the [Ru(bpy)_3_]^2+^ luminophore and the [Ir(sppy)_3_]^3−^ complex are labeled as Ru^2+^ and Ir^3−^, respectively. Adapted with permission from [56], Copyright (2024) Royal Society of Chemistry

This mechanism is generally observable at high Ru(bpy)_3_^2+^ concentration (hundreds µM to mM) [54], barely showing any practical interest for analytical purpose. However, recent investigations of homogeneous oxidation of TPrA by a freely diffusing Ir complex demonstrated that this strategy can increase the ECL signal from a microbeads immunoassay, i.e., heterogeneous system by a “redox mediated” pathway (Fig. 6) [55, 56]. The Ir(III) complex oxidized at the electrode to produce Ir(II) can oxidize the TPrA, which result in the formation of TPrA^•^, and in addition, it reacts with Ru(bpy)_3_^+^ to generate the excited state of Ru(bpy)_3_^2+^ with the overall effect of increasing the ECL signal up to 107%.

### ECL mechanism by “reductive-oxidation”

This category comprises the coreactants that after reduction form a high energetic radical that oxidize the luminophore, mainly peroxides such as persulfate [13, 57, 58], hydrogen peroxide [59], and benzoyl peroxide [60].

**Scheme 5:**
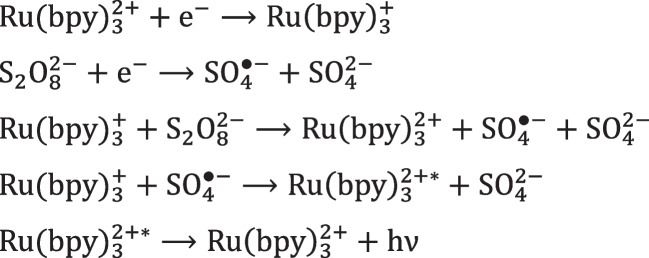
ECL “reductive-oxidation” mechanism of S_2_O_8_^2–^ and Ru(bpy)_3_^2+^

All these coreactants follow the same reaction pathway, as illustrated for peroxydisulfate in Scheme 5.

The striking difference between S_2_O_8_^2–^ and TPrA is that an heterogeneous mechanism is not possible and the reduction of the Ru(bpy)_3_^2+^ to Ru(bpy)_3_^+^ is essential. This has been demonstrated experimentally by labeling Ru(bpy)_3_^2+^ on microbeads, and the imaging analysis by a microscope did not reveal any ECL emission when the S_2_O_8_^2–^ was the coreactant [61]. This further confirms the great importance of using TPrA for the ECL imaging of cells or large biological entities [62–64].

### ECL from luminol

**Scheme 6:**
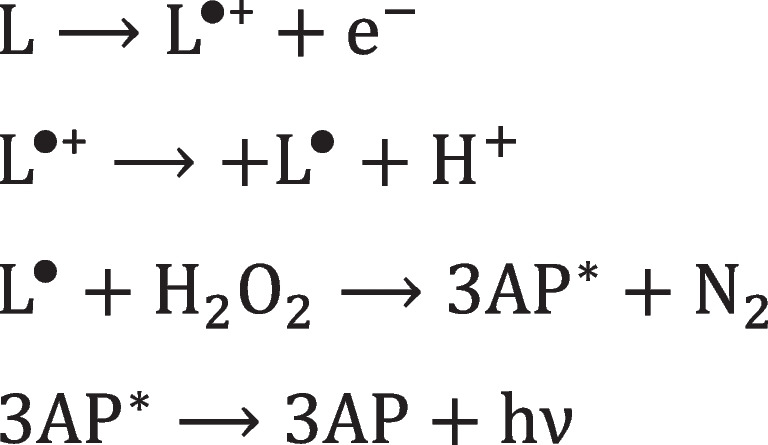
Simplified reaction mechanism for ECL of luminol (L) with H_2_O_2_

Luminol is an organic molecule which application in ECL is directly taken from chemiluminescence (CL) [65, 66]. Luminol has a characteristic chemiluminescence emission centered at 425 nm, from the 3-aminophthalatedianion (3AP) excited state, though depending on the solvent can emit light in a range from around 424 to 510 nm, with ECL centered at 440 nm [33, 67]. Luminol reacts with H_2_O_2_ to generate CL in the presence of a suitable catalyst (peroxidase or metal ions), while in ECL, the electrochemical stimulus triggers the light emission. For this reason, luminol is used in combination with oxidases which produce H_2_O_2_ in analytical applications. The mechanism proceeds by luminol oxidation at the electrode and following reaction with H_2_O_2_. The adduct releases a N_2_ molecule to form the 3AP in the excited state that emits light (Scheme 6). Depending on the electrode material, solvent, and pH, the ECL emission from luminol is observed generally in the potential range between 0.3 and 0.5 V (vs. Ag/AgCl) [68–70].

In conclusion, we have to distinguish between sensor applications and ECL investigation on mechanisms and signal enhancement. If the target is the development of a sensor for a specific analyte, there is no doubt that the couple Ru(bpy)_3_^2+^/TPrA offers superior advantages in terms of ECL intensity, high reproducibility, simplicity, and the opportunity to use the heterogeneous ECL approach to implement a real sensing device. On the other hand, still the Ru(bpy)_3_^2+^/TPrA ECL mechanism needs further investigation toward fundamental understanding [15, 52], and in particular, to increase the ECL signal intensity [54, 55]. Moreover, other new ECL systems can be introduced [35, 71], in particular, concerning the luminophore, but these have to demonstrate true advantages when compared with the Ru(bpy)_3_^2+^/TPrA ECL system.

## Electrode materials

Electrode materials for ECL does not differ from general electroanalytical techniques, although some particular attention has to be taken when the electrode material affects the ECL mechanisms, to match it with the ECL system of interest [72].

### Noble metals: platinum and gold

Pt and Au electrodes are widely used in electrochemistry, as well as in ECL, in particular, for organic solvents and aprotic conditions and for the characterization of new luminophores, for example. This condition enables a potential window of about 3 V, enough to explore a wide range of annihilation mechanisms. Metals can have high purity (up to 99.99%); the shape is highly reproducible and the ductility allows the fabrication of electrodes with different dimensions (diameter from cm to µm). For example, with ultramicroelectrodes, the adverse effects of ohmic drop and cell time constant on establishing electrode potential, which originates from the fast potential switch in annihilation ECL, can be minimized. Another advantage of metal electrodes is their superior resistance to fouling when compared to glassy carbon.

Some drawbacks arise when metal electrodes are employed in aqueous solution, a general environment for sensing applications. The small overpotential for hydrogen or oxygen evolution reaction makes these reactions to compete with luminophore or coreactant electrochemical reactions.

**Fig. 7:**
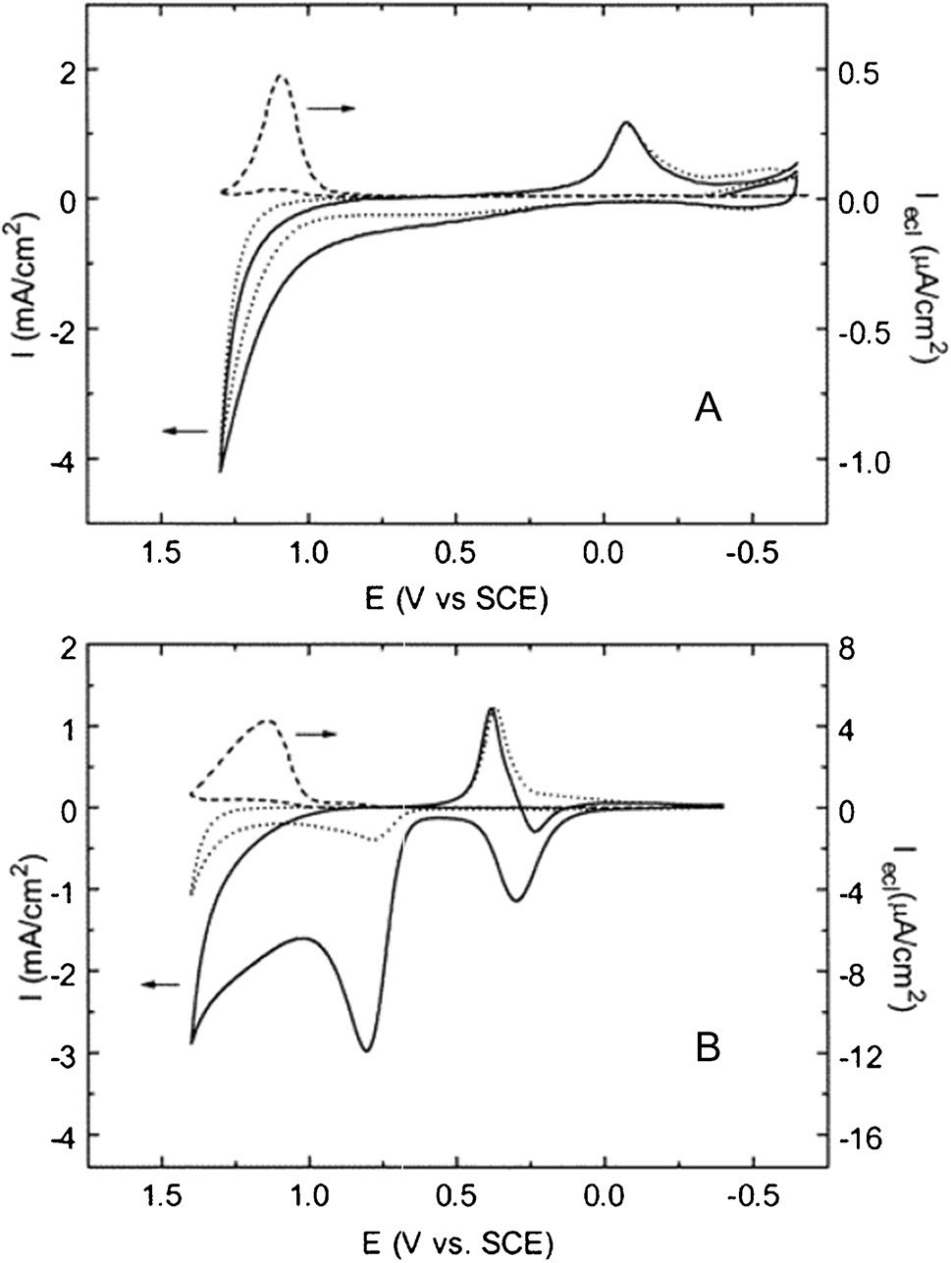
Cyclic voltammogram and ECL curve at (**a**) platinum and (**b**) gold electrodes in 0.15 M phosphate buffer solutions (pH 7.5) containing 100 mM TPrA and 1 µM Ru(bpy)_3_^2+^. The dotted line represents data in the absence of both Ru(bpy)_3_^2+^ and TPrA. Potential scan rate, 0.1 V s^−1^. Adapted with permission from [73], Copyright (2000) American Chemical Society

Concerning the use of TPrA, its oxidation corresponds with the generation of an oxide layer on the electrode surface (0.2∼1.4 V vs. SCE) that prevents almost completely the TPrA oxidation reducing the heterogeneous electron transfer reaction. This results in serious quenching of the ECL signal. Au oxide formation is approximately 400 mV more positive than Pt oxide that gives Au a 10 times higher ECL emission compared to Pt (Fig. 7) [73].

Generally, the addition of surfactants, also alkanethiols in case of Au, could alleviate this problem by the formation of a hydrophobic layer on the electrode surface that reduces the generation of the oxide and increases the heterogeneous electron transfer of TPrA oxidation [74]. Another drawback encountered with noble metal electrodes can be the adsorption of intermediates originated from the coreactant oxidation that leads to poisoning of the metallic electrode surface. Therefore, either surfactants are used or not, the surface of metal electrodes requires a cleaning procedure (mechanical or electrochemical) after the ECL measurement to restore its initial state.

Gold electrodes were initially employed in the first commercially available ECL instrument (Origen I analyzer) introduced by IGEN International in 1994 [48, 49]. However, in contemporary technology for immunoassay detection, Roche Diagnostics utilizes Pt instead [75]. This is mainly due to the lower propensity of Pt to form metal oxides and its easier electrochemical cleaning process [44].

### Carbon-based electrodes

The first difference for these electrodes comes with the carbon hybridization. Generally, carbon electrodes are based on carbon black, carbon nanotubes, graphene, graphite, and glassy carbon, therefore sp^2^ carbon. However, diamond (sp^3^ carbon) electrodes are also employed in electrochemistry, and ECL as well, which will be discussed in a following section. Carbon electrodes show a fast kinetics of TPrA heterogeneous electron transfer reaction, and a sluggish kinetic for hydrogen and oxygen evolution, if compared to metal electrodes [76]. This makes carbon electrodes particularly suitable for ECL generation in aqueous electrolyte, and biosensors development, although the physical stability restricts the application to disposable platforms. This was excellently demonstrated by Meso Scale Discovery with the ECL analyzer based on multiarray technology where the platform for the ECL immunoassay (screen-printed carbon ink electrodes) resembles the ELISA method, where the recognition element is bound on the bottom of a well microtiter plates, and a sandwich immunoassay is formed by a secondary antibody labeled with the luminophore [6].

**Fig. 8:**
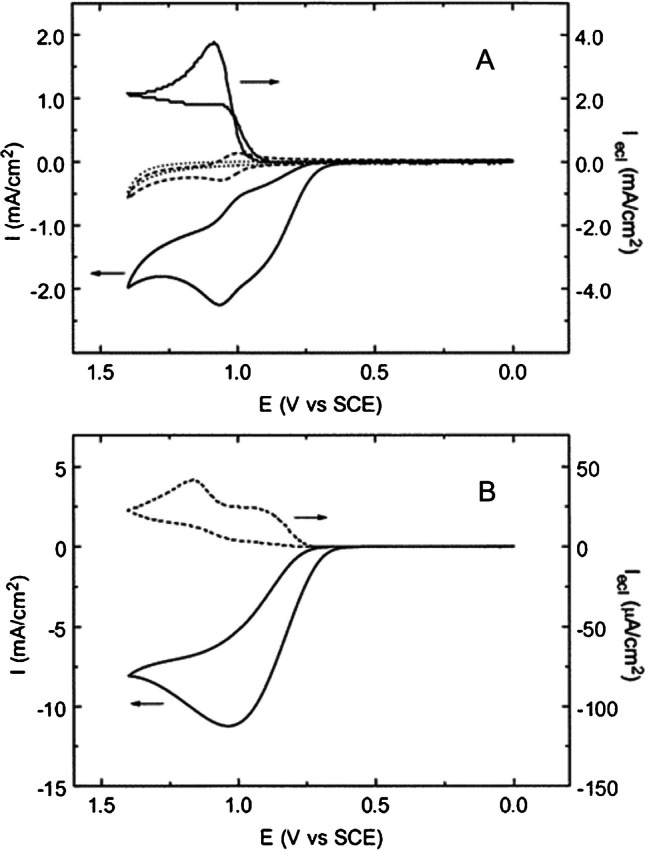
(**a**) Cyclic voltammograms and ECL curve of 1 mM Ru(bpy)_3_^2+^ at a glassy carbon electrode in 0.15 M phosphate buffer solution (pH 7.5) in the presence (solid line) and absence (dashed line) of 10 mM TPrA. The dotted line represents data in the absence of both Ru(bpy)_3_^2+^ and TPrA. (**b**) Cyclic voltammogram and ECL curve at a glassy carbon electrode in 0.15 M phosphate buffer solution (pH 7.5) containing 100 mM TPrA and 1 µM Ru(bpy)_3_^2+^. Potential scan rate, 0.1 V s^−1^. Adapted with permission from [73], Copyright (2000) American Chemical Society

When compared to Au and Pt, glassy carbon showed 10 times and 100 times higher ECL emission, respectively (Fig. 8) [73].

In analogy with metal electrodes, glassy carbon requires a cleaning procedure (mechanical or electrochemical) after the ECL measurement to restore its initial state, because anodic oxidation during ECL generation and adsorption of intermediates lead to signal decrease [77]. In addition to glassy carbon, other carbon allotropes have been used for ECL, namely carbon nanotubes and graphene which generally require a substrate that serves as support [37]. These carbon materials also permit to prepare optically transparent electrodes for special applications, such as imaging, biosensors, or spectroscopy [62, 78–80].

Carbon nanomaterials not only act as electrode material but can be scaffolds to bind the recognition unit of biosensors, for example antibodies [81] or enzymes [34, 37]. Nevertheless, the main disadvantage of carbon electrodes can be the quality of the carbon structures. In fact, sp^3^ carbon or defects in the sp^2^ carbon structure could cause generation of unspecific signal that increases the background.

Although not strictly carbon electrodes, conducting polymers can be used for ECL. For example, poly(3,4-ethylenedioxythiophene) (PEDOT) and polypyrrole (PPy) films, and their composites, have been used with the luminol/H_2_O_2_ system for electrical property characterization of polymers by ECL imaging [69, 82, 83].

A particular carbon allotrope which is diamond is an intrinsic semiconductor with an indirect band gap of 5.47 eV [84]. To acquire the metallic conductivity necessary for electrochemistry, it is generally doped with boron at concentration around or higher than 10^20^ [B] cm^−3^ [85]. The electrochemistry of boron-doped diamond (BDD) has been established with application in chemical and biochemical sensing, environmental remediation, electrosynthesis, electrocatalysis, and energy storage and conversion [86], and recently, BDD has also been applied successfully to ECL [51]. Well-known characteristics include wide potential window in aqueous electrolyte (low catalytic activity for oxygen and hydrogen evolution reaction), low capacitive current, and physical and chemical stability.

**Fig. 9:**
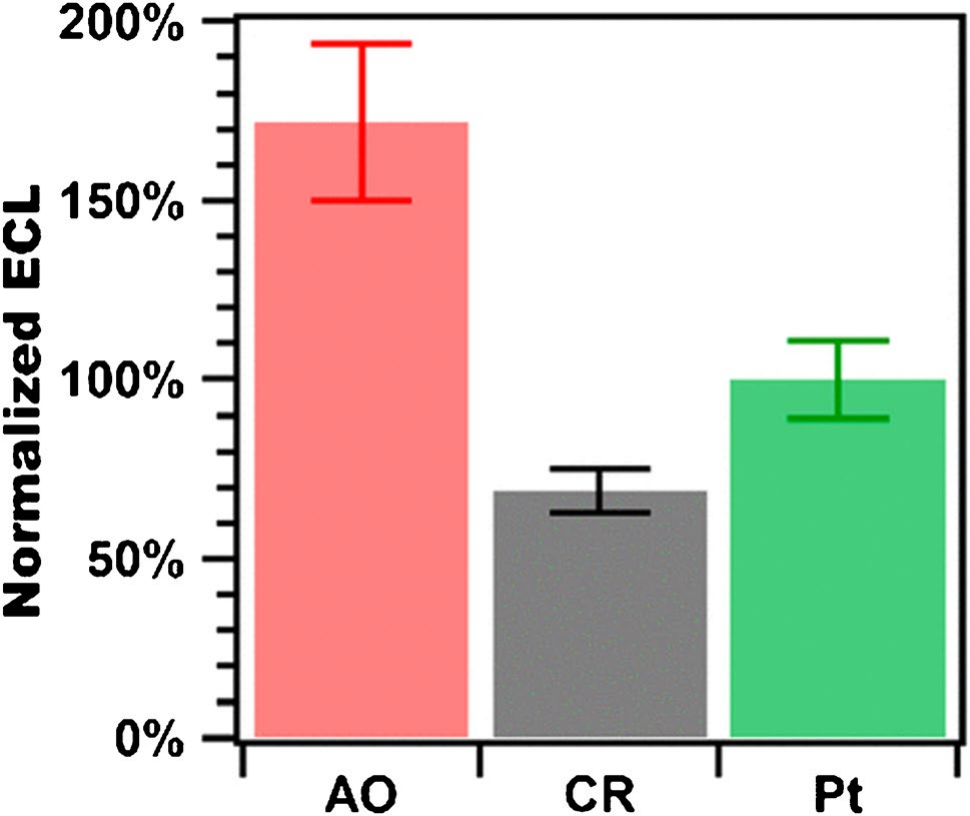
Integrated ECL from Ru(bpy)_3_^2+^-labeled beads for anodic-oxidized (AO) BDD (red), cathodic-reduced (CR) BDD (gray), and Pt (green) electrodes. Adapted with permission from [87], Copyright (2022) American Chemical Society

ECL at BDD electrodes has been evaluated for the Ru(bpy)_3_^2+^/S_2_O_8_^2−^ system where it showed higher emission than glassy carbon thanks to the low activity for hydrogen evolution [58]. For the Ru(bpy)_3_^2+^/TPrA systems, BDD showed higher emission in the heterogeneous system with microbeads compared to conditions mimicking the commercial immunoassay with Pt electrode (Fig. 9) [87].

The peculiar properties of BDD enabled to produce directly in situ the coreactant for the Ru(bpy)_3_^2+^ from sulfate or carbonate oxidation which generates S_2_O_8_^2–^ or H_2_O_2_, respectively, for the “reductive-oxidation” ECL mechanism [88, 89]. The generation of the H_2_O_2_ coreactant directly in situ from carbonate oxidation was also reported for luminol ECL [33].

Generally, optimal boron doping level is around 1% of B/C ratio (≈ 2 × 10^21^ [B] cm^−3^) for most of the common ECL systems [90].

### Transparent electrodes

These electrodes find applications in combination with microscopy to image processes limited to the surface proximity, particularly for imaging of biological samples. Generally, two types of electrodes are available: glass/metal oxides (indium-doped tin oxide or fluorine-doped tin oxide) and carbon (nanotubes or graphene) on glass or polymer support. However, the electrochemistry of these two types of electrode is quite different, for example, the TPrA oxidation reaction rate on a carbon nanotubes electrode is about 30 times faster than on indium-doped tin oxide electrode, with an estimated heterogeneous electron transfer constant of 2.6 × 10^−2^ cm s^−^^1^ and 8 × 10^−^^4^ cm s^−^^1^, respectively [78].

### Paper-based microfluidic and screen-printed electrodes

**Fig. 10:**
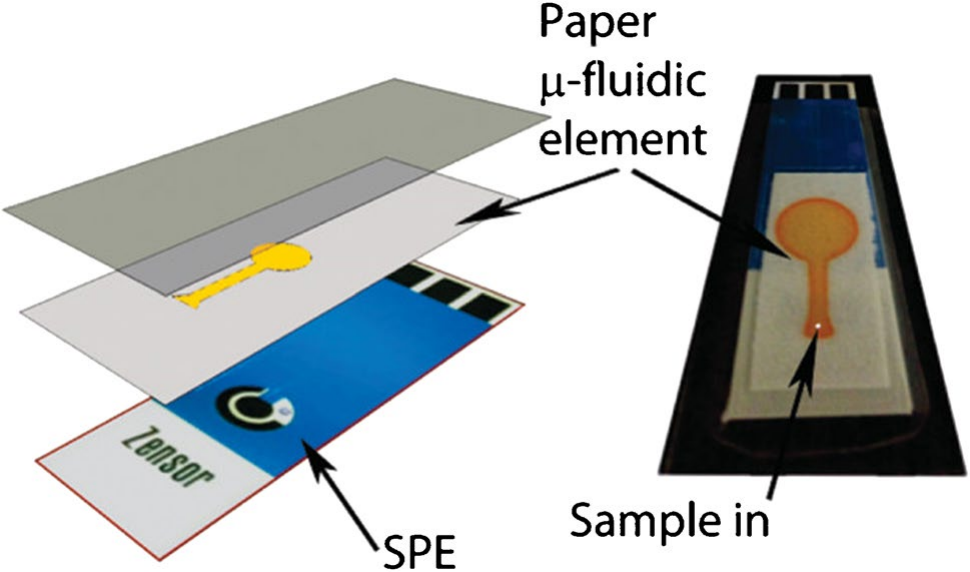
Example of coupling paper-based microfluidic and screen-printed electrode to realize an analytical device. Adapted with permission from ref [92], Copyright (2011) American Chemical Society

Microfluidic paper-based analytical devices (mPADs) represent a class of materials that can be successfully coupled with ECL detection [91]. Inkjet printing technology can be employed to fabricate paper microfluidic substrates, or alternatively using the strip-based rapid test, that in combination with screen-printed electrodes create a simple, easy to use, and disposable sensors. Moreover, the ECL signal can be detected by a smartphone camera to develop a portable device (Fig. 10) [92, 93].

## ECL protocols

In recent years, the research of advanced analytical techniques has been triggered by the need for sensitive detection methods in various fields, particularly in strategies for biomarker detection. Consequently, a lot of effort has been dedicated to the development of new ECL-based protocols for the accurate determination of specific analytes through immunoassays, enzyme-based assays, and other methodologies, further harnessing the potential of ECL in modern analytical chemistry.

### Luminophore conjugation

**Fig. 11:**
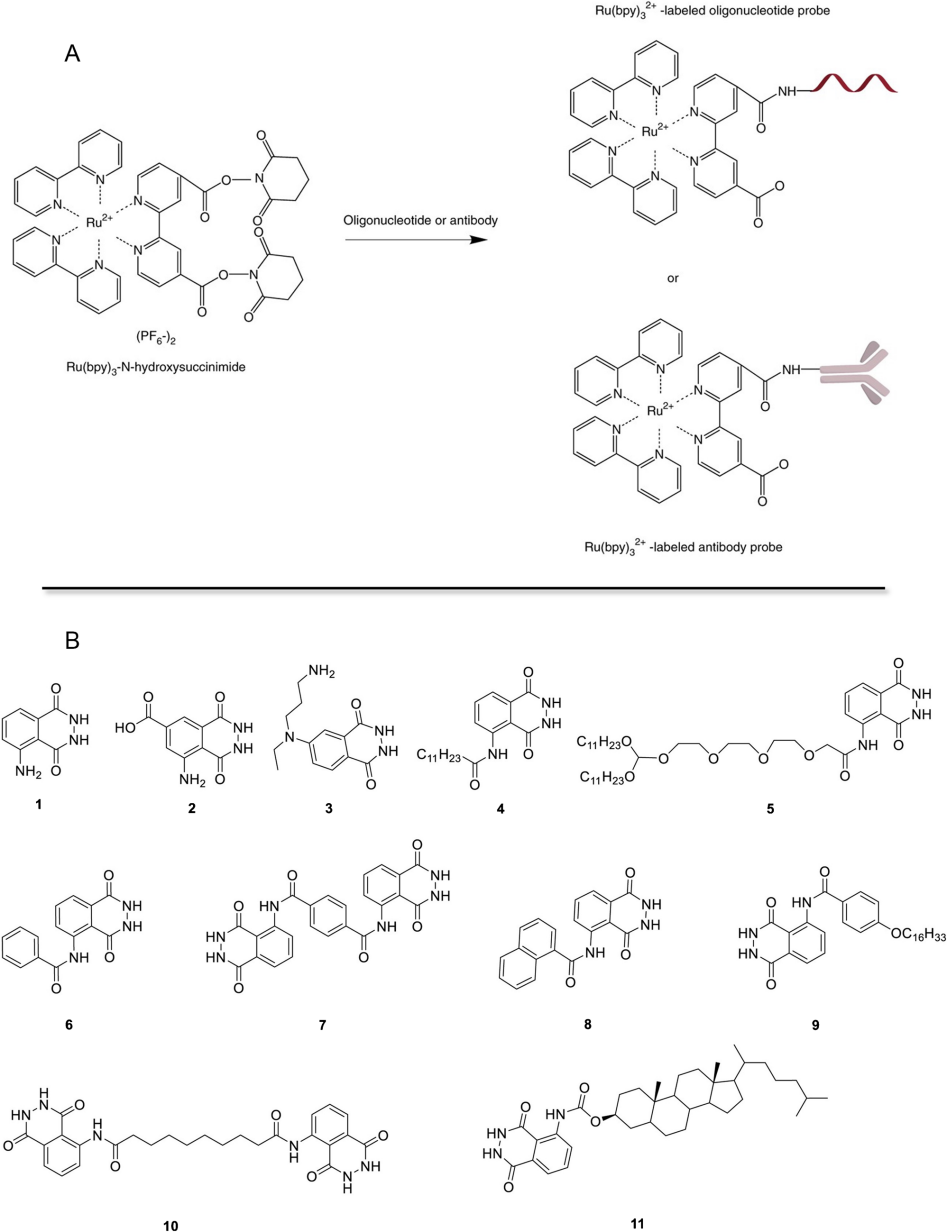
(**a**) Synthetic route for Ru(bpy)_3_^2+^-labeled antibody and Ru(bpy)_3_^2+^-labeled oligonucleotide probes through NHS ester. Adapted with permission from [95], Copyright (2014) Nature Journal. (**b**) Structures of luminol and luminol derivatives. Adapted with permission from [97], Copyright (2023) Elsevier

The transition metal complex Ru(bpy)_3_^2+^ is one of the most luminophore used in ECL, renowned for its widespread utilization as a probe. Ru(bpy)_3_^2+^ /TPrA systems react effectively and emit light in aqueous medium at room temperature (∼25 °C) and at the proper pH range (see Fig. 5) in the presence of dissolved oxygen and other impurities [94]. Ru(bpy)_3_^2+^ can function as a reporter molecule through its ability to form conjugates with reactive groups present within biomolecules. Derivatives of the metal complex containing functional groups facilitating conjugation are employed for this purpose enabling the coupling with amino groups in proteins, peptides, ligands, and synthetic oligonucleotides. For instance, antibodies can be effectively labeled by covalently attaching the amino group in lysine to a Ru(bpy)_3_^2+^ -NHS ester, elucidating its applicability in immunoassays and beyond (Fig. 11) [95]. Moreover, the synthesis, labeling, and bioanalytical applications of this tris(2,2′-bipyridyl)ruthenium(II)-based ECL probe demonstrate its versatility and significance in modern analytical chemistry [94, 95, 96, 97, 98].

On the other hand, luminol is the most used organic ECL luminophores, featuring a low excitation potential and high ECL efficiency across both aqueous and solid phases [70, 94, 96]. Luminol is widely used in immunoassays, small molecule detection, and enzyme activity analyses. Its versatility is also given by the presence of an active amino group on its aromatic ring, facilitating facile coupling to other materials, particularly biomacromolecules, via covalent bonds preserving the stability of ECL signals and enhancing the accuracy of detection systems. Other luminol derivatives with different chemical functionalities, introduced onto the aromatic ring, were investigating to further enhance the ECL efficiency and improve the physicochemical properties of luminol-based assays (Fig. 11) [97]. For example, by adding a hydrophilic carboxylate group to the benzene ring of luminol, the resulting m-carboxy luminol overcame the solubility limitations of its parent compound in neutral solutions [99]. Moreover, incorporating other chemical functionalities could further facilitate coupling with biological probes [100]. These modifications could accelerate the advancement and utilization of luminol systems in various bioanalytical applications, such as ECL-based cellular imaging and the in vivo detection of biomarkers.

### ECL Immunoassay

ECL immunoassays (ECLIAs) are one of the most important methods of accurate and sensitive biomarker detection, and thus are widely used for clinical diagnosis and have been successfully implemented in commercialized devices [15, 98]. Labeled immunoassays have witnessed significant advancements, with various label reagents like chromogenic, fluorescent, chemiluminescent (CL), biochemiluminescence (BCL), electrochemical, and ECL being extensively explored [101]. However, challenges persist with certain methods: fluorescent immunoassays are hindered by the fluorescence of proteins themselves, while enzyme-labeled immunoassays face issues related to enzyme instability and the relatively low bioaffinity of large enzyme-labeled antibodies. Additionally, electrochemical immunoassays are constrained by their limited reproducibility and sensitivity.

There are mainly two configurations in ECLIA. In one, immunoreactions are typically conducted on the surface of an electrode and categorized into sandwich, competitive, and direct approaches. Among these methodologies, sandwich ECLIA has been widely favored due to its excellent performance, characterized by high sensitivity, selectivity, and a broad linear range.

In an alternative approach, immunoreactions typically occur in solution rather than on the surface of the working electrode, while ECL measurement takes place on either a bare or chemically modified working electrode in the presence of a coreactant. This approach relies on signal variations arising from steric hindrance, conformational changes, and alterations in the diffusion coefficient induced by the immunoreactions in solution. This type of ECLIA offers simplicity and ease of automation owing to its non-separation process but face the potential adverse effects of complex matrices.

**Fig. 12:**
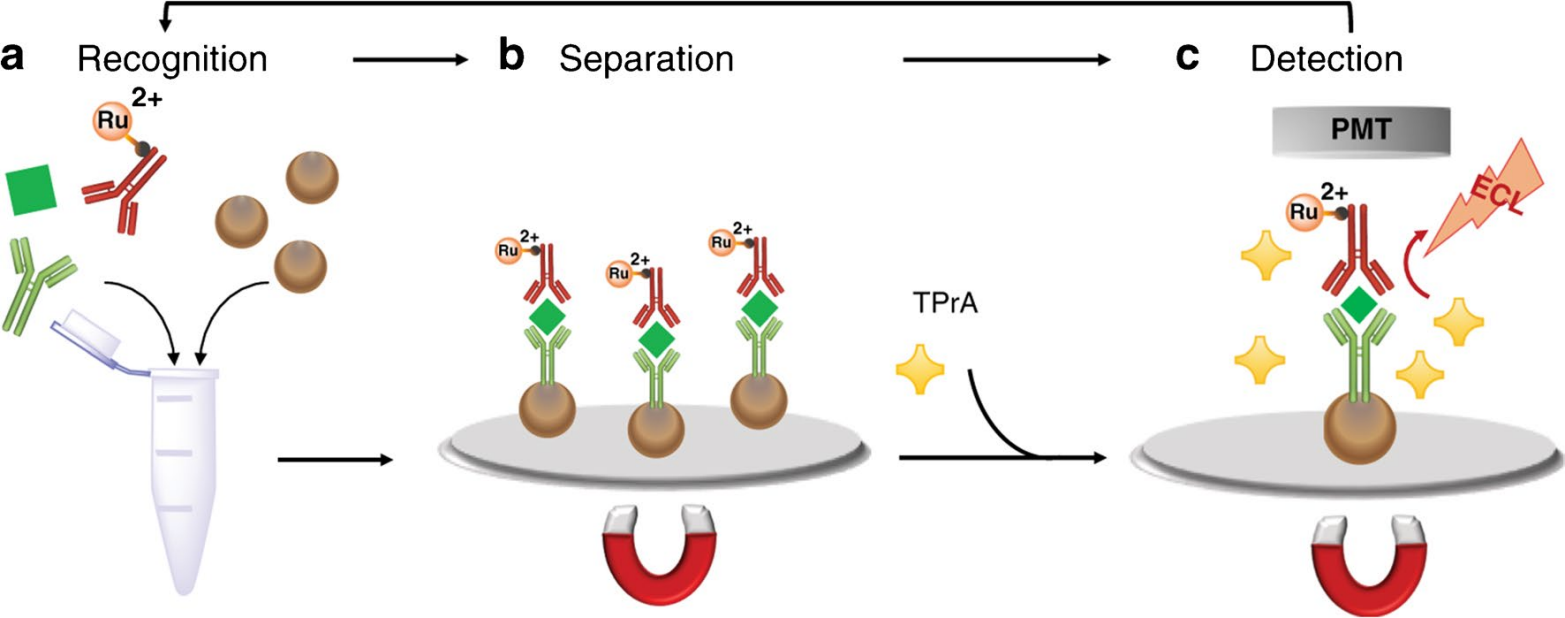
Schematic representation of the commercial ECLIA system showing (**a**) the recognition phase where the immune sandwich labeled with biotin on one side and the ECL luminophore on the other, is formed and bind to streptavidin-coated microbeads. (**b**) The microbeads separation through a magnetic field and (**c**) the detection of ECL emission by a photomultiplier tube (PMT). Adapted with permission from [15], Copyright (2020) Nature Journals

An important ECLIA that is performed on bare working electrodes using immunomagnetic beads is the automated ECL immunoassay system (Elecsys) commercialized by Roche Diagnostic and using Ru(bpy)_3_^2+^ and the TPrA system [15]. These immunomagnetic beads, featuring specific capture probes immobilized on their surface (referred to as MBs), play a crucial role in efficiently preconcentrating targets from complex samples [102, 103]. Subsequently, the ECL immunocomplexes formed on the surface of the MBs can be easily separated from the excess ECL probe under through an external magnetic field (Fig. 12).

### ECL-DNA biosensing

ECL methods have been employed in nucleic acid assays, leveraging the Watson–Crick base-pairing rule. Typically, the target single-stranded DNA (ss-DNA) is detected using a sandwich hybridization format. In this format, the target ss-DNA first hybridizes with capture probes immobilized on a surface and subsequently hybridizes with ECL probes in solution [104, 105]. Various hybridization strategies have been developed for DNA hybridization ECL biosensing, such as structure switching, target-induced strand displacement, and superhybridization. One straightforward approach is the structure switching format (DNA nanoswitch) [106], where the target single-stranded DNA (ss-DNA) hybridizes with an ECL reagent-labeled hairpin immobilized on the electrode surface leading to a change in the ECL signal [104, 107]. An alternative method for detecting ss-DNA is a label-free one, where the ECL reagent (usually Ru(phen)_3_^2+^) interacts with the double-stranded DNA (ds-DNA) [102, 108].

### Enzymatic ECL biosensing

**Fig. 13:**
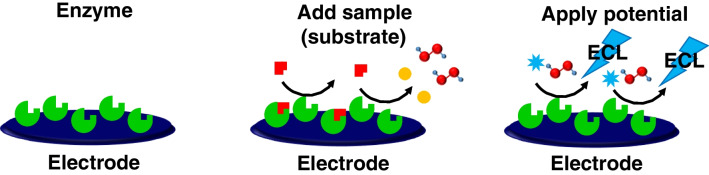
Schematic representation of an enzymatic ECL biosensor pathway. Adapted with permission from [34], Copyright (2022) Wiley

Enzymatic ECL biosensing involves an enzyme catalytic reaction with ECL detection, where coreactants serve as either a coproduct or a cofactor of an enzymatic reaction [34] (Fig. 13).

The enzyme is typically immobilized on the electrode surface by physical entrapment or chemical methods forming covalent (e.g., cross-linking with glutaraldehyde or carbodiimide) or non-covalent (e.g., adsorption, affinity) chemical bonds. The immobilization strategy ultimately affects the performance of the biosensor because it can affect the catalytic function and the shelf-life of the enzyme.

Common examples of enzymatic ECL systems include the luminol-H_2_O_2_ system and the Ru(bpy)_3_^2+^-β-nicotinamide adenine dinucleotide (NADH) system as follows:

$$\begin{array}{cc}{\text{Substrate}}_\text{Red}+{\text{O}}_2\longrightarrow{\text{Substrate}}_\text{Ox}+{\text{H}}_2{\text{O}}_2&(\mathrm{oxidase})\end{array}$$

$$\begin{array}{cc}{\text{H}}_2{\text{O}}_2+\text{Luminol}\longrightarrow\text{ECL}&(\mathrm{applied}\;\mathrm{potential})\end{array}$$

$$\begin{array}{cc}{\text{Substrate}}_\text{Red}+{NAD}^+\longrightarrow{\text{Substrate}}_\text{Ox}+\text{NADH}&(\mathrm{dehydrogenase})\end{array}$$

$$\begin{array}{cc}\text{NADH}+{Ru\left(bpy\right)}_3^{2+}\longrightarrow\text{ECL}&({\mathrm{applied}\;\mathrm{potential}})\end{array}$$

In the luminol-H_2_O_2_ system, H_2_O_2_ is produced through the interaction of dissolved oxygen with enzymatic substrates, catalyzed by the oxidase. Subsequently, H_2_O_2_ couples with the electrochemical oxidation of luminol, leading to the generation of excited 3-aminophthalic acid. This system has primarily been utilized in quantifying the concentration of enzymatic substrates, including glucose, uric acid, cholesterol, and ethanol, among others. Additionally, it has been applied in ECL immunoassays, using antibodies labeled with glucose oxidase as ECL probes for the identification of biomarkers of interest [109, 110]. However, it is essential to mitigate the chemiluminescence (CL) background arising from the catalytic reaction of metal ions in this system.

In the Ru(bpy)_3_^2+^-NADH system, NADH coenzyme acts as a coreactant, generated by NAD+-dependent enzymes such as glucose dehydrogenase, alcohol dehydrogenase, and lactate dehydrogenase using specific substrates. This setup has been employed in quantifying the concentration of enzymatic substrates like glucose, alcohol, and lactate. Nonetheless, these enzymes have drawbacks, including susceptibility to activity loss and high costs.

## Conclusion

In conclusion, ECL stands out as a promising avenue for analytical applications. Throughout this review, our aim has been to provide a comprehensive guide to utilize ECL in analytical contexts, elucidating the roles of different components in ECL generation mechanisms and strategies for optimization.

When considering luminophores, inorganic complexes offer distinct advantages in terms of functionalization and homogeneity. On the other hand, organic molecules pose challenges due to issues related to water solubility and conjugation synthesis. Nanomaterials, while holding promise, face challenges in standardization, which impacts the accuracy and reproducibility of results.

Classification of ECL systems based on reaction mechanisms provides insight into optimization strategies. Additionally, the choice of electrode material plays a crucial role in sensitivity, with Pt and Au electrodes preferred in organic solvents, and carbon electrodes offering advantages in aqueous environments. Although, Pt electrode is also used in commercial analyzers.

The recent rise in the utilization of microfluidic paper-based analytical devices reflects a trend toward simplicity and disposability, thereby enhancing accessibility to ECL technology.

Finally, we have discussed new ECL-based protocols that offer opportunities for precise analyte determination through techniques such as immunoassays, enzyme-based assays, and DNA capturing probe systems, leveraging the conjugation of luminophores on specific probes.

In summary, this review sheds light on the multifaceted aspects of ECL in analytical applications, covering fundamental mechanisms as well as emerging protocols. We believe these insights will prove valuable to researchers in the field.
